# Sustainable Re-Use of Brewer’s Spent Grain for the Production of High Protein and Fibre Pasta

**DOI:** 10.3390/foods11050642

**Published:** 2022-02-23

**Authors:** Francesca Cuomo, Maria Carmela Trivisonno, Silvio Iacovino, Maria Cristina Messia, Emanuele Marconi

**Affiliations:** 1 Department of Agricultural, Environmental and Food Sciences (DiAAA), Università degli Studi del Molise, Via De Sanctis, 86100 Campobasso, Italy; francesca.cuomo@unimol.it (F.C.); mariacarmela.trivisonno@unimol.it (M.C.T.); s.iacovino@studenti.unimol.it (S.I.); marconi@unimol.it (E.M.); 2 Università Campus Bio-Medico di Roma, Via Alvaro del Portillo 21, 00128 Roma, Italy

**Keywords:** brewer’s spent grains, by-products, dry pasta, high-protein, high-fibre

## Abstract

Brewer’s spent grains are one of the principal by-products of the brewing industry. For protein and fibre content, this by-product represents an interesting raw material to be reused for manufacturing many other products. To maximize the nutritional characteristics of this by-product, in this study, ingredients derived from brewer’s spent grains were included in the design of innovative dry pasta. Two brewer’s spent grains derivative ingredients, one enriched in proteins and the other in fibre were blended with semolina. Based on the rheological evaluation, the optimal amount of the two ingredients for producing pasta was determined. In particular, pasta responding to the claims “*High Protein*” and “*High Fibre*” was realized using the formulation enriched with 15% of protein-rich ingredient and the claim “*High Fibre*” and “*Source of proteins*” using the formulation enriched with 10% of fibre-rich ingredient. The final products were compared to 100% semolina and 100% wholegrain semolina pasta for composition, color, texture, and cooking quality, revealing excellent quality characteristics. The newly formulated pasta represents a successful match of technological aptitude, nutritional/sensorial quality, and sustainability.

## 1. Introduction

The reduction in waste and the reuse of by-products are the basis of the circular economy that finds opportunities in regeneration and recycling of waste materials and energy that become inputs to other processes to build a virtuous closed-loop system that minimizes waste, pollution, and carbon emissions [1,2].

Recently, the European Commission has developed a plan within the European Green Deal named “Farm to Fork strategy” that, among other aspects, aims at developing a more sustainable food system, from production to consumption [3]. Within the agri-food system, there are numerous examples of by-products rich in nutrients, micronutrients, or other potentially interesting molecules. For instance, in the past, Marconi et al. [4] used by-products from the barley pearling process for making functional pasta enriched in dietary fibre and β-glucans. Considering other barley transformation processes, one noteworthy by-product of the brewing sector are the spent grains. Brewer’s spent grains (BSG) are made of residual insoluble malted barley, comprising the external sheets of the seed such as the coat-pericarp and the husk layers [5]. According to recent reports [6], the worldwide annual production of beer is 1.9 billion hectoliters and considering that about 20 kg of BSG is produced for every beer hectoliter, a production of around 39 million tons of BSG is esteemed, 3.4 million tons of which are from the European Union. In the last few years, the upcycling of BSG has become a widespread practice for the multiple possibilities of reuse as a sorption material [7,8,9,10], and as a fuel after proper treatments [11] for use in animal [11,12,13,14] and human nutrition [15,16,17,18]. The high interest in these by-products is currently making BSG a profitable raw matter for several industrial fields [19]. The interest in BSG originates from its composition. Indeed, it is a good source of lipids, proteins, and most of all, fibre—being rich in cellulose, hemicellulose, and lignin and phenolic compounds. On the other hand, the high moisture content of BSG could represent one point against its upcycling since can be subjected to microbial spoilage, and would be difficult to move this resource to other processing sites [20]. In a recent report, Cimini and Moresi [21] reported that the costs for drying BSG greatly increase the price of the dried product with respect to analogous conventional lignocellulose residues. Nevertheless, several strategies are currently applied for drying BSG using cost-effective and energy-saving processes [22]. In particular, this approach yields effective results when factories produce a high volume of BSG by-products that are converted into nutrient-rich ingredients for a wide variety of foods including beverages, bread, pizza crust, pasta, granola bars, meat alternatives, and smoothies [23].

In the few last years, various research based on the reuse of BSG for human nutrition have been published. BSG were, indeed, included in the formulation of meat sausage (Frankfurters) [24], in the recipe of extruded snacks [18,25,26], for producing bread [17,27] and cookies [28]. In all these studies, the BSG inclusion caused fibre enrichment in the composition of the final products.

Among cereal-based foods, pasta represents a pillar of the Mediterranean diet because it is characterized by a high content of complex carbohydrates with a low glycemic index [29]. Pasta generally contains proteins at levels of about 12% but this value can be enhanced through different strategies [30,31]. Innovative pasta recipes, including the replacement of semolina with alternative ingredients and non-traditional raw materials, have already been proposed [31,32,33,34,35,36,37,38].

A few years ago, Cappa and Alamprese [39] included BSG in fresh egg pasta to obtain a fibre-enriched product. To improve the pasta structure, they also included egg white powder in their pasta formulation.

Very recently, Nocente and coworkers [16] used a blend of semolina and BSG for producing long dry pasta. BSG-enriched pasta was characterized by a high content of fibre and antioxidants. The same authors used BSG from the einkorn and tritordeum brewing processes for producing short pasta [15] realizing also in that last study a product enriched in fibre. 

In this regard, Sahin and colleagues [5] added BSG rich in protein and rich in fibre also for producing fresh pasta, and the two ingredients were added to obtain a final pasta that could comply with the nutritional claim’s source of fibre or high fibre according to the European Community Regulation No. 1924/2006 [40].

Finally, Schettino and colleagues [41] used bioprocessed BSG, treated with xylanase and fermented by *Lactiplantibacillus plantarum*, to obtain fortified semolina pasta (macaroni shape) labeled as “*High fibre*” and “*Source of protein*” product. 

The goal of the present study was to include BSG derivatives in the production of “*High-protein*” and “*High fibre*” dry pasta, with excellent structural and sensorial characteristics. The produced pasta was evaluated for nutritional and textural features and cooking quality.

## 2. Materials and Methods

### 2.1. Materials

Brewer’s spent grains rich in protein and fibre were supplied by EverGrain (Leuven, Belgium). BSG were stabilized through drying (low water activity). The dry powder was then milled and air-classified with a patent-pending process. These two mechanical steps allow to reduce the particle size of the BSG, as well as to partially separate the protein and fibre fractions. The resulting fractions were commercialized under the name EverVita Pro and EverVita Fibra (here indicated with EVP and EVF ingredients, respectively).

The proximal composition and particle size of the ingredients are shown in Table 1. 

Durum wheat semolina and wholegrain semolina were purchased from a local distributor.

### 2.2. Chemical and Nutritional Analysis

Moisture, lipid, and ash were determined according to ICC methods 109/1, 136, and 104/1, respectively [42]. Protein content was determined through a Leco nitrogen determiner, model FP 528 (Leco Corp., St. Joseph, MI, USA) according to the Dumas combustion method, AACC method 46-30.01 [43] (Nx6.25). Dietary fibre was determined according to AACC Method 32-05.01 [43]. Arabinoxylans were determined according to Hashimoto et al. [44]; β-glucans were determined according to the AACC Method 32-23.01 [43].

Amino acids were analyzed after acidic hydrolysis [31]. Briefly, a sample, corresponding to 25 mg of protein was hydrolyzed with 25 mL of 6 N HCl at 110 °C for 24 h. Afterwards, the sample was cooled, filtered, dried, and suspended in 0.1 N HCl. Before analysis, all samples were diluted 1:50–1:100 with ultra-pure water and analyzed by the ICS6000 chromatographic system (Thermo Fisher Scientific S.p.A, Milano, Italy). Separation was performed with an Aminopac PA10 analytical column (250 × 2 mm, 8.5 μm particle size) (Thermo Fisher Scientific S.p.A, Milano, Italy). 

The chemical score (CS) was calculated according to Food and Agriculture Organization [45] using the recommended amino acid scoring pattern for older children, adolescents, and adults. The amino acid that has the lowest chemical score within the amino acid pattern is referred to as the limiting amino acid. The chemical score is given by the ratio between the amount of a given essential amino acid in one gram of the protein in the food matrix and the amount of the same amino acid in one gram of the reference protein.

All used reagents were of analytical grade and were purchased from Sigma-Aldrich (Milan, Italy).

### 2.3. Rheological Determinations

Farinograph and alveograph analyses were carried out according to AACC International Methods (54-21.01 and 54-30.02 [43], respectively). For mixtures with EVP and EVF, some adjustments to the Chopin method were applied to finally obtain an Alveograph response. The outcomes of the farinograph analysis were used to establish the amount of water to add for the alveoghraph test. The practice of adapting the hydration levels is commonly applied for studying flours that require more water to be tested with the alveoconsistograph [46], such as the case of flours with high protein, or high fibre content or starch with different amylose/amylopectin ratios [47]. 

### 2.4. Formulations 

Formulations were prepared by mixing EVP and EVF ingredients with semolina. EVP and EVF ingredients were added in increasing amounts, EVP from 10 to 20% and EVF from 5 to 10%. The formulations that gave the best response in terms of nutritional and rheological/technological performances together with the achievement of the nutritional claims “*High fibre*” and “*High protein*” (according to EU Regulation 1924/2006 [40]), were selected for being used in the pasta making process. 

### 2.5. Pasta Making

**Figure 1:**
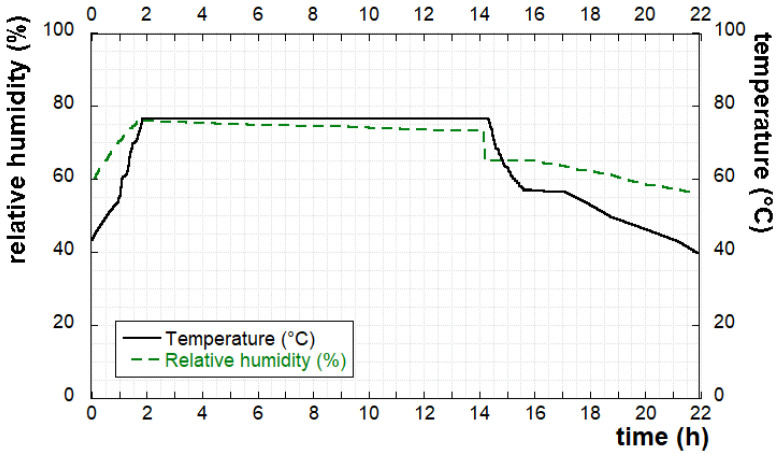
Pasta drying diagram: relative humidity and temperature vs. time.

Spaghetti was manufactured through an experimental pasta-making semi-industrial plant (NAMAD, Roma, Italy) composed of a press and a dryer, following AACC Method 66-41.01 [43]. The press, with a working capacity of 5–20 kg, was equipped with: (1) a vacuum mixing and extruding system composed of a screw-barrel that pushes the product, (2) a die that restricts the flow-out of the dough, and (3) a water-cooling jacket of the barrel and the extrusion head to reduce heat and to maintain a constant extrusion temperature lower than 50 °C. Each series of spaghetti was dried at a temperature lower than 80 °C using temperature/humidity equilibrium curves. An example of the adopted drying diagram is shown in Figure 1.

The dryer was equipped with: (a) a ventilator unit to ensure uniform temperature and ventilation in all parts of the apparatus; (b) a moisture regulation and control unit; and (c) a regulation and program unit equipped with a computer and dedicated software developed on the LabView platform by DRD AUTOMAZIONE srl (San Giovanni in Galdo, Campobasso, Italy). 

Spaghetti was produced using the operative parameters reported in Table 2. 

Bronze (brass/bronze alloy) dies with Teflon and bronze inserts were used for producing spaghetti with a mean diameter of 1.7 and 1.8, respectively. Extrusion occurred at 30 °C ± 2. EVF-enriched pasta, pasta 100% semolina-2, and 100% wholegrain semolina were both made with a Teflon-coated die. Pasta enriched with EVP was made using bronze and Teflon dies, indicated with EVP15-2(B) and EVP15-2(T), respectively.

### 2.6. Color

The color was measured using CIE (Commission Internationale de l’Eclairage) L*, a* and b* color system, where L* describes brightness, a* is redness, and b* is yellowness. Color measurements were performed in triplicate with a colorimeter model CR300 (Minolta Italia, S.p.A., Milan, Italy).

### 2.7. Cooking Quality

Optimal cooking time (OCT) was determined according to International Standard ISO 7304-1 [48], by observing the time of disappearance of the central core of the spaghetti strand during cooking (every 30 s), by squeezing the spaghetti between two transparent glass slides.

Sensorial attributes such as firmness, liveliness, and starch release were evaluated according to the standard ISO 7304-1 [48]. The panel was composed of eight trained assessors that, for each sensory characteristic, assigned a score from 0 to 100 (high values correspond to positive qualities; low values to negative qualities). The combination of the scores of the individual characteristics determines the total score of the cooking quality. A score ranging from 10 to 100 was used to determine cooking quality. Spaghetti with a total score of ≤40 was classified as of poor or mediocre quality; >40 to ≤50—not completely satisfactory; >50 to ≤70—fair; >70 to 80—good; and >80—excellent. 

### 2.8. Texture Analysis

The texture analysis was carried out using a TAXT2 Texture Analyzer (Stable Micro Systems, Godalming, England) interfaced with a PC. Firmness was measured through a blade probe (HDP/LKBF). The preparation of the samples was carried out according to AACC Approved Method 66–50.01 [43]. Tensile strength was measured through a spaghetti probe (A/SPR). For measuring tensile strength, cooked spaghetti was located through slots in the parallel friction rollers and then wound around two or three times to reduce any slippage and also to anchor the sample ends. The rollers ensure that the sample does not split or cut during the test and that the break occurs along the extended part of the sample. Tensile strength is the force at the sample break. Flexure was measured on uncooked spaghetti through a spaghetti flexure probe (A/SFR). 

### 2.9. Statistical Analysis

SPSS software (version 23.0, IBM SPSS Statistics, Armonk, NY, USA) was used for statistical analysis. All determinations were made in triplicate and were expressed as the mean value ± the standard deviation. Analysis of variance (ANOVA) and Tukey HSD tests were performed and differences at *p* < 0.05 were considered as significant.

## 3. Results and Discussion

Table 3 compares different BSG products/by-products/derivatives, used for food production reported in the literature. Considering the data of protein and fibre content and those relative to particle size (where available), it arises that the lower the BSG particle size was, the higher the protein content was, particularly when the BSG-based cereal was barley. Sahin et al. [5,27] used two BSG-derivative ingredients, one rich in protein, EVP, with a particle size of about 100 µm, and the other EVF, rich in fibre with a particle size of about 300–500 µm. The present study is based on BSG-derivative ingredients obtained from the same provider as Sahin et al. [5,27], but with some differences in particle size. The EVP ingredient here used, indeed, is characterized by a particle size of around ~52 µm which is smaller than that used by Shain and colleagues. As for the protein content, it is slightly lower than the latter but higher compared to the other BSG found in the literature. EVF used here presents a particle size comparable to the mean values of other BSG.

### 3.1. Composition and Rheological Evaluation of Blends 

The designed formulations made of semolina and BSG-derivative ingredients developed to meet dietary/nutritional and technological/rheological aspects suitable for producing pasta are reported in Table 4. Blends were enriched in protein and fibre with the increasing of EVP and EVF ingredients. 

The content of β-glucans was low (around 0.15% for EVF and around 0.20% for EVP) since beer production is realized using barely genotypes with low β-glucans content and because during malting these compounds are degraded by β-glucanase [49].

The addition of EVP and EVF to semolina caused important modifications to the rheological properties of the resulting dough. At the beginning of the investigation, EVP/semolina and EVF/semolina blends were formulated using a semolina with a normal protein content (Semolina-1). The composition of the mixtures enriched with 10, 15, and 20% EVP (EVP10, EVP15, and EVP20, respectively) and 5 and 10% EVF and (EVF5 and EVF10, respectively) is reported in Table 4. A significant increase in the water adsorption Farinograph index is observed in all the blends containing the EVP and EVF ingredients compared to Semolina-1 and the same was observed for the developing time of dough, except for the EVF5 formulation that had a developing time similar to dough 100% Semolina-1 and 100% wholegrain semolina. Values of water absorption of the blends were more similar to that of the wholegrain semolina. The increasing of water adsorption, as well as the lengthening of dough developing time, can be explained by the high content of protein and fibre (arabinoxylan). For this reason, the alveograph test of EVP10, EVP15, EVP20, and EVF10 required 14, 20, 26, and 12% more water, respectively, than the quantity required by the conventional Chopin method that usually works at constant hydration conditions. The results of the alveograph test showed a significant decrease in W (except for EVF5 due to the lower level of semolina substitution) compared to Semolina-1. This indicated that dough resistance to deformation decreased, in EVF10, EVP10, EVP15, and EVP20 mixtures because of the diluting and weakening of the gluten network. The increase in the P/L ratio in the same formulation indicated that dough was more compact and less elastic. Similar results were found by Pasqualone et al. [50] who investigated the rheological properties of blends of re-milled semolina and durum wheat by-products micronized and air-classified. 

Based on the rheological characteristics, dough developed by the EVP20 formulation was not suitable for pasta making. Subsequently, aiming at obtaining pasta with a maximum level of EVP of 15% and with “*High protein*” claim [40], semolina with higher protein content (14.9% f.w.) (Semolina-2) was selected and used for successive pasta-making processing. The rheological properties of Semolina-2 and the EVP blend with Semolina-2 (EVP15-2) are shown in Table 4.

Water adsorption and dough developing time gave analogous results to those observed in blends with Semolina-1, while values of W from the Alveograph test (for EVP15-2 20% more water was added) were similar for Semolina-2 and EVP15-2. On the other side, EVF was used at levels of 10% with Semolina-1 because these conditions ensured reaching the characteristics of “*High fibre*” [40] pasta.

### 3.2. Pasta Characterization 

Proximate composition and color analyses outcomes are reported in Table 5. EVP-enriched pasta presented a protein content of about 18% and of fibre higher than 8%. With respect to the proteins, EVP-enriched pasta would provide about 30% of the protein intake recommended by EFSA [51] for an adult of 75 kg (RDA 0.83 g/kg body weight), instead of a common pasta that would provide about 20%. 

In addition, EVP15-2 pasta reached the claim “*High protein*” according to the European Regulation n. 1924/2006 on nutrition and health claims on food [40], i.e., at least 20% of the energy of EVP15-2 derives from proteins. As for fibre, considering 100 g of pasta, EVP15-2 and EVF10 formulations can provide approximately 35% and 32% of the dietary fibre intake per day, respectively, according to the EFSA [52]), more than thrice the amount provided by 100% semolina pasta (RDA 25g/day). The fibre content of EVP15-2 and EVF10, moreover, corresponded to the “*High fibre*” claim of the European Regulation n. 1924/2006 [40]. For the protein content, EVF10 corresponded to the “*Source of protein*” nutritional claim [40].

The amino acid composition of proteins is an important factor in determining the quality (biological value) of proteins. From the results reported in Table 6, it appears that, as known, the limiting amino acid of semolina and therefore of the reference pasta is lysine. Semolina and whole meal semolina pasta have a chemical score of 43 and 50, respectively. In EVF and EVP ingredients, the limiting amino acid is still lysine with a slightly higher chemical score of 73 and 71, respectively. In the pasta that derives from the blends of BSG-derived ingredients, the limiting amino acid remains, obviously lysine, but compared to semolina, the chemical scores are slightly higher, equal to 52 and 46 in EVP15-2 and EVF10 pasta, respectively, denoting an improvement in the biological value of proteins.

EVF and EVP-enriched spaghetti had similar arabinoxylans content (around 4.5%), higher than semolina pasta (2.5%), but lower than in wholegrain pasta (6.3%). 

As for color analysis, pasta enriched with EVP and EVF showed a significant reduction in the L* (brightness) parameter, an expected effect considering that the ingredients were darker in color. The L* reduction was higher for pasta produced with EVP and with Teflon-coated die than for EVP15-2(B) and EVF10 that was more similar to wholegrain semolina spaghetti. The redness value, a*, was lower for semolina pasta compared to all the other pasta. Contrarily, the yellow index gave an opposite trend with higher values associated with Semolina-2 and lower values in the following order: semolina > EVF10 > EVP15-2(B) > EVP15-2(T). Nocente et al. [16] recorded equal color characteristics for pasta produced with the addition of BSG to their formulations. 

**Figure 2:**
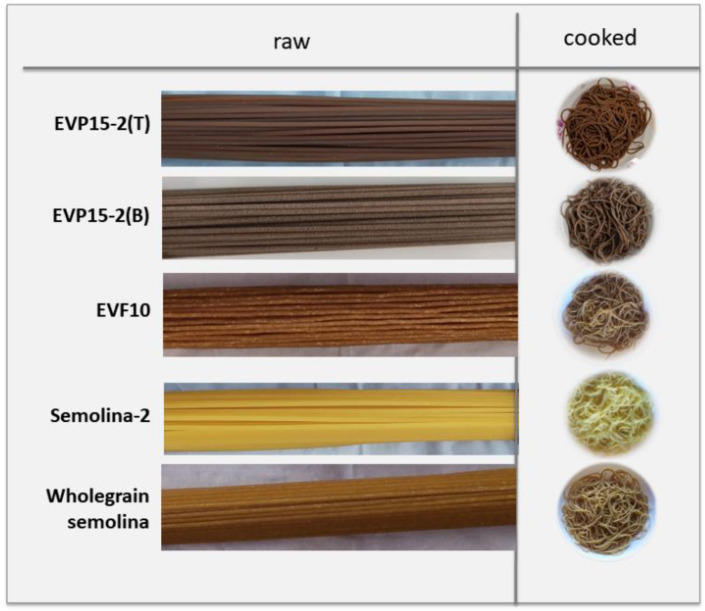
Pictures of dried (on left) and cooked (on right) spaghetti.

The color of raw and cooked pasta is evident from the images shown in Figure 2. As can be observed from the image, EVF10 presented a rough surface probably due to the non-homogeneous particle size. This aspect and the presence of white dots (that disappeared after cooking) on the surface of EVF-enriched spaghetti was, indeed, ascribed to the larger particle size of EVF (320 µm) powder with respect to EVP (52 µm). By comparing Teflon-extruded spaghetti, it is evident that the smaller particle size of the EVP derivative allowed the ingredient to be better included in the structure of pasta that presented a smoother surface compared to EVF10.

### 3.3. Cooking Quality and Texture Analysis 

As shown in Table 7, the OCT of pasta EVP15-2(T) was similar to semolina pasta. EVF10 was produced with Semolina-1 and presented a shorter OCT compared to pasta produced with EVP and Semolina-2 blends. EVP15-2(B), semolina, and wholegrain semolina spaghetti, on the other hand, presented a longer cooking time compared to EVP15-2(T). Yoshino and coworkers [53] studied the water sorption characteristics of spaghetti prepared using different dies and found that in spaghetti produced through a bronze die the kinetics of water adsorption was faster than in those produced with a Teflon die due to the higher porosity given by the bronze die. Thus, the higher OCT recorded in EVP15-2(B) can be ascribed to the higher diameter that was around 1.8 mm in EVP15-2(B) compared to 1.7 mm for EVP15-2(T) and semolina pasta.

As for the cooking quality aspects, firmness was evaluated positively for pasta made with the addition of EVP and EVF ingredients and was similar to the firmness of semolina and wholegrain semolina pasta. Scores relative to starch release or stickiness were slightly higher for high-protein pasta, compared to EVF10 and semolina spaghetti, regardless of the type of die used and were significantly higher than the score assigned to 100% wholegrain semolina spaghetti. The starch release score assessed to EVP15-2(T) was comparable to those of semolina and wholegrain semolina pasta. Verardo et al. [54] also found that pasta produced by replacing 50% semolina with a 45% coarse fraction of air-classified barley flour and 5% of vital gluten was characterized by high protein content and absence of stickiness. Liveliness showed a trend mirroring that seen for starch release with higher scores for EVP15-2(T) and EVP15-2(B). Overall, pastas prepared with EVP ingredients as well as semolina pasta received *Excellent* as score, while wholegrain semolina pasta was of good quality. 

The characteristics of firmness, evaluated through instrumental analysis, were well related to sensorial analysis and very close to each other. Tensile strength values of spaghetti prepared using Teflon die or bronze die were similar. Other research [55,56] found that bronze die pasta was characterized by a higher breaking strength than Teflon die pasta which seemed to disagree with our outcomes. Nevertheless, in the works cited above [55], they measured the breaking strength through a three-point bending test where pasta is tested in the raw state while our result was obtained on cooked spaghetti.

As reported in Table 7, the breaking force values were lower in Teflon die spaghetti produced with EVF and EVP, compared to 100% semolina pasta. This can depend on the ingredients interfering with the formation of the gluten network [57,58]. As a result, protein-enriched spaghetti was less elastic and broke more easily. Bronze die spaghetti, nevertheless, presented higher values of flexure than Teflon die pasta, which could be explained by the higher diameter of the former (1.82 mm vs. 1.70 mm).

Collectively, this study confirmed that the use of raw materials different from semolina in pasta making modifies the rheological dough characteristics and affects the technological performances [59]. Spaghetti realized in the present study have higher cooking quality and improved textural characteristics, compared to others produced through BSG enrichment. Nocente et al. [16], indeed, produced spaghetti with poor firmness, bulkiness, and stickiness evaluated both by means of sensorial and textural analysis. The characteristics were probably affected by the particle size of BSG and the low temperature used for the drying process. To improve the structural properties of fresh-egg pasta (lasagne) proposed by Cappa and Alamprese [39], the addition of egg white powder was necessary to valorize BSG inclusion in the preparation, unless the mechanical properties were poor. BSG-enriched pasta proposed by Schettino and colleagues [41] presented higher hardness and lower chewiness values improved by the bioprocessing of BSG that probably contributed to favor the distribution of BSG proteins in the pasta structure. A comparison with the long pasta produced by Shanin et al. [5] cannot be made, regarding cooking quality and textural characteristics because they realized pasta with a lab-scale extruding system and did not dry the product, conditions that were different from those adopted in our investigation. 

As for the nutritional aspects, here, we have proved that pasta with EVP ingredients can reach the nutritional claims “*High protein*” and “*High fibre*” [40], while long [16] and short [15] shaped dry pasta by Nocente and colleagues improved only the total dietary fibre content. Fresh-egg pasta by Cappa and Alamprese [39] also improved their formulation for the fibre content, while Schettino et al. [41] found their best formulation corresponded to the nutritional claims of “*High fibre*” and “*Source of protein*” [40], (i.e., 12% of the energy of the product is provided by proteins). The same nutritional claims were achieved by Shanin and coworkers [5]. Overall, pasta produced in this study matches well with the recent food trends. EVP and EVF-enriched pasta, indeed, join: (i) the regeneration and recycling of waste materials, which are at the basis of the virtuous system of the circular economy; (ii) the market demand of people who care a great deal about environmental issues and/or with special nutritional needs.

## 4. Conclusions

The present study demonstrated that BSG-derivative ingredients enriched in protein and in fibre were successfully exploited to develop new pasta with added value in terms of nutritional and technological quality characteristics. The rheological characteristics of the EVP/semolina and EVF/semolina blends revealed that the addition of the ingredients to dough determined the necessity to adjust some parameters for dough formation that reflected also on the pasta making process. 

EVP and EVF long pasta were successfully produced using a Teflon-coated die and a bronze die, showed excellent cooking quality, and achieved the characteristics for being labeled as “*High protein*” and “*High fibre*” pasta. Overall, the results of this research show that including BSGs in the formulation of pasta implies the advantage of adding onto an already saturated market, an innovative product that contains a valuable industrial by-product. In particular, the ingredients here used, represent a stable and sustainable solution for BSG upcycling.

## Figures and Tables

**Table 1:** Composition (dry weight, d.w.) of EVP and EVF ingredients.

	Protein (% dw)	Fibre (% dw)	Fat (% dw)	Carbohydrates (% dw)	Ash (% dw)	β-glucans (% dw)	Arabinoxylans (% dw)	Size, d90 (µm)	Moisture (%)
EVP	32.2	38.7	15.6	10.5	3.1	0.15	15.5	52	2.4
EVF	22.8	49.6	5.9	10	3.8	0.20	20.0	320	3.0

EVP:EverVita Pro; EVF: EverVita Fibra.

**Table 2:** Operative parameters for pasta production.

Pasta Formulation	Blend Powder Moisture %	Added Water %	Extrusion Process Parameters		
			Screw Speed (rpm)	Vacuum Pressure (bar)	Head Pressure (bar)
EVP15-2(T)	12.5	40.0	35.2	0.9	56.0
EVP15-2(B)	12.5	39.5	35.2	0.9	75.0
EVF10	12.9	33.0	35.2	0.9	93.0
Semolina-2	14.2	28.0	35.2	0.9	112.0
Wholegrain semolina	13.6	31.0	35.2	0.9	74.0

EVP15-2(T), pasta from blend 85% Semolina 2 and 15% EVP extruded with Teflon-coated die; EVP15-2(B), pasta from blend 85% Semolina 2 and 15% EVP extruded with bronze die; EVF10, pasta from blend 90% Semolina 1 and 10% EVF extruded with Teflon-coated die; PS, pasta from Semolina 2 extruded with Teflon-coated die; PWS, pasta from Wholegrain semolina extruded with Teflon-coated die.

**Table 3:** Protein and fibre content (% d.w.), particle size and treatment and products realized using different BSG.

BSG Cereal		Protein	Fibre	Particle Size	BSG Treatment	Product	Reference
Einkorn Tritordeum		32.5 21.6	30.5 25.9	≤700 µm	Storing at −20 °C, drying at 60 °C for 72 h and micronization	Pasta	[15]
Barley		14.5	51.3	≤700 µm	Storing at −20 °C drying at 60 °C for 72 h and micronization	Pasta	[16]
Barley		22.4	67.9	n.a.	n.a.	Bread	[17]
Barley		20.3	53.4	n.a.	Refrigeration, drying at 150 °C for 4 h and milling at 0.5 mesh screens	Bread	[18]
Barley		20.3	53.4	n.a.	Refrigeration, drying at 150 °C for 4 h and milling at 1, 0.5 and 0.25 mesh screens	Ready-to-eat snacks	[25]
BSG-derivative ingredients Barley	EVP EVF	36.8 23.4	46.8 65.8	100 µm 300–500 µm	Drying, milling and fractionation	Bread	[27]
BSG-derivative ingredients Barley	EVP EVF	36.8 23.4	46.8 65.8	100 µm 300–500 µm	Drying, milling and fractionation	Fresh pasta	[5]
Barley + Rice		23.2 25.8 31.2	75.3 66.2 62.4	425–850 µm 212–425 µm <212 µm	Drying at 45 °C for 24 h in oven, milling and sieving (sieves 850, 425 and 212 µm)	Frankfurter	[24]
Barley		27.0	57.0	<500 µm	Freeze-drying and milling	Barley snacks	[26]
Barley		n.a	n.a	<500 µm	Vacuum over drying at 60 °C for 48 h and milling	Fresh-egg pasta	[39]
Barley + Corn	Bioprocessed BSG	21.0	60.8	250–500 µm 500–750 µm 750–1000 µm	Enzymatic treatment, fermentation, freeze-drying and milling	Pasta	[41]
BSG-derivative ingredients enriched in protein and fibre Barely + Corn	EVP EVF	32.2 22.8	38.7 49.6	52 µm 320 µm	Drying, milling and fractionation	Pasta	This study

n.a = not available.

**Table 4:** Moisture, protein, fibre, and arabinoxylans content of semolina and EVP and EVF blends and results from Farinograph and Alveograph tests. Different letters in rows indicate statistically significant differences (*p* < 0.05).

	EVP10	EVP15	EVP20	EVF5	EVF10	Semolina 1	Wholegrain Semolina	EVP15-2	Semolina 2
Moisture (%)	13.2 ± 0.1 ^b^	12.5 ± 0.2 ^a^	12.2 ± 0.1 ^a^	14.0 ± 0.2 ^c,d^	13.8 ± 0.1 ^c,d^	14.2 ± 0.1 ^d^	13.6 ± 0.1 ^b,c^	12.5 ± 0.2 ^a^	14.2 ± 0.2 ^d^
Protein (Nx6.25) (% d.w.)	17.7 ± 0.1 ^c^	18.4 ± 0.1 ^d^	19.3 ± 0.1 ^e^	15.9 ± 0.2 ^a^	16.5 ± 0.1 ^b^	15.6 ± 0.1 ^a^	16.8 ± 0.1 ^b^	19.9 ± 0.1 ^f^	17.4 ± 0.1 ^c^
Fibre (% d.w.)	7.2 ± 0.3 ^c^	9.7 ± 0.4 ^e^	9.7 ± 0.2 ^e^	5.6 ± 0.1 ^b^	8.8 ± 0.2 ^d^	3.7 ± 0.3 ^a^	8.7 ± 0.3 ^d^	9.7 ± 0.2 ^e^	3.5 ± 0.3 ^a^
Arabinoxylans (% d.w.)	3.9 ± 0.1 ^b,c^	4.5 ± 0.3 ^d^	5.2 ± 0.2 ^e^	3.5 ± 0.2 ^b^	4.3 ± 0.1 ^c,d^	2.6 ± 0.2 ^a^	6.1 ± 0.3 ^f^	4.4 ± 0.2 ^d^	2.4 ± 0.1 ^a^
β-glucans (%d.w.)	0.15 ± 0.01 ^a,b^	0.16 ± 0.005 ^a,b^	0.17 ± 0.004 ^b,c^	0.15 ± 0.0 ^a,b^	0.15 ± 0.003 ^a,b^	0.14 ± 0.01 ^a^	0.35 ± 0.01 ^d^	0.19 ± 0.02 ^c^	0.16 ± 0.005 ^a,b^
Farinograph parameters:									
Water adsorption (%)	62.5 ± 0.8 ^d^	64.4 ± 0.4 ^e,f^	65.1 ± 0.3 ^f,g^	58.6 ± 0.2 ^b^	60.3 ± 0.4 ^c^	56.7± 0.5 ^a^	63.8 ± 0.3 ^e^	65.5 ± 0.4 ^g^	58.7 ± 0.2 ^b^
Dough development time (min)	6.6 ± 0.4 ^c^	15.9 ± 0.2 ^d^	18.8 ± 0.4 ^e^	4.0 ± 0.1 ^a^	6.2 ± 0.3 ^c^	4.2 ± 0.2 ^a^	3.8 ± 0.1 ^a^	19.0 ± 0.2 ^e^	5.3 ± 0.3 ^b^
Stability (min)	17.0 ± 0.2 ^e,f^	16.4 ± 0.3 ^e^	17.2 ± 0.2 ^f,g^	17.8 ± 0.3 ^g^	17.5 ± 0.2 ^f,g^	10.9 ± 0.1 ^b^	4.3 ± 0.2 ^a^	15.6 ± 0.3 ^d^	14.6 ± 0.5 ^c^
Degree of softening (UF)	2 ± 0.2 ^a^	20 ± 3.0 ^d^	45 ± 2 ^e^	15 ± 1.1 ^c^	6 ± 0.5 ^b^	20 ± 1 ^d^	42 ± 1.2 ^e^	43 ± 3.0 ^e^	20 ± 1.0 ^d^
Alveograph parameters:									
P (mm)	108 ± 4 ^b,c,d^	117 ± 5 ^c,d,e^	98 ± 12 ^b^	147 ± 3 ^f^	75 ± 2 ^a^	102 ± 5 ^b,c^	128 ± 7 ^e^	122 ± 5 ^d,e^	126 ± 9 ^e^
L (mm)	32 ± 2 ^c^	19 ± 1 ^b^	10 ± 1 ^a^	42 ± 1 ^d^	49 ± 3 ^d^	75 ± 3 ^e^	31 ± 5 ^b^	18 ± 1 ^b^	85 ± 6 ^f^
P/L	3.4 ± 0.1 ^b^	6.2 ± 0.2 ^c^	9.6 ± 1.5 ^e^	3.5 ± 0.2 ^b^	1.5 ± 0.1 ^a^	1.4 ± 0.1 ^a^	4.1 ± 0.7 ^b^	7.7 ± 0.5 ^d^	1.5 ± 0.1 ^a^
W (10^−4^ J)	144 ± 12 ^c,d^	101 ± 3 ^b^	44 ± 3 ^a^	245 ± 11 ^e^	137 ± 13 ^c^	252 ± 6 ^e^	159 ± 8 ^d^	115 ± 3 ^b^	116 ± 11 ^b^

EVP10, blend of 90% semolina 1 + 10% EVP; EVP15, blend of 85% semolina 1 + 15% EVP; EVP20, blend of 90% semolina 1 + 20% EVP; EVF5, blend of 95% semolina 1 + 5% EVF; EVF10, blend of 90% semolina 1 + 10% EVF; Semolina 1, 100% semolina 1 (protein content 15.6% (Nx6.25)); Wholegrain semolina, 100% wholegrain semolina; EVP15-2, blend of 85% semolina 2 + 15% EVP; Semolina 2, 100% semolina 2 (protein content 17.4% (Nx6.25)).

**Table 5:** Proximate composition (g/100 g fw), achievement of nutritional claims diameter and color characteristics of different pasta. Different letters in rows indicate statistically significant differences (*p* < 0.05).

Pasta	EVP15-2(T)	EVP15-2(B)	EVF10	Semolina-2	Wholegrain Semolina
Moisture	10.4 ± 0.2 ^a^	10.1 ± 0.3 ^a^	10.2 ± 0.1 ^a^	10.0 ±0.2 ^a^	10.5 ± 0.1 ^a^
Protein	17.8 ± 0.3 ^c^	18.1 ± 0.2 ^c^	14.6 ± 0.1 ^a^	15.4 ± 0.2 ^b^	15.0 ± 0.1 ^a,b^
Fibre	8.7 ± 0.4 ^b,c^	8.5 ± 0.5 ^b,c^	7.9 ± 0.6 ^b^	3.1 ± 0.1 ^a^	7.6 ± 0.2 ^b^
Fat	3.5 ± 0.3 ^d^	3.2 ± 0.2 ^c,d^	2.0 ± 0.1 ^b^	1.5 ± 0.1 ^a^	2.9 ± 0.3 ^c^
Ash	1.7 ± 0.1 ^b^	1.7 ± 0.1 ^b^	1.6 ± 0.1 ^b^	1.2 ± 0.3 ^a^	1.7 ± 0.2 ^b^
Carbohydrate *	57.9 ± 0.6 ^a^	58.9 ± 0.7 ^a^	64.1 ± 0.5 ^b^	69.5 ± 0.2 ^c^	63.2 ± 0.2 ^b^
Arabinoxylans	4.5 ± 0.2 ^b^	4.6 ± 0.2 ^b^	4.5 ± 0.4 ^b^	2.5 ± 0.2 ^a^	6.3 ± 0.3 ^c^
Nutritional Claims [40]:					
High protein ^1^	YES	YES			
High fibre ^2^	YES	YES	YES		YES
Source of protein ^3^			YES	YES	YES
Source of fibre ^4^				YES	
Pasta diameter (mm)	1.7 ±0.03	1.82 ± 0.02	1.7 ± 0.4	1.70 ± 0.03	1.70 ± 0.02
Color (as is):					
L*	34.5 ± 0.9 ^a^	42.8 ± 0.9 ^b^	44.9 ± 1.3 ^b^	57.1 ± 1.5 ^c^	44.9 ±1.2 ^b^
a*	7.3 ± 0.2 ^e^	3.4 ± 0.2 ^b^	6.4 ± 0.2 ^d^	-4.5 ± 0.6 ^a^	4.9 ± 0.2 ^c^
b*	12.6 ± 0.2 ^a^	13.3 ± 0.7 ^a^	25.5 ± 0.4 ^b^	34.7 ± 0.4 ^d^	29.9 ± 0.3 ^c^
Color (ground pasta):					
L*	45.7 ± 1.2 ^a^	46.2 ± 0.8 ^a^	51.5 ± 0.7 ^b^	56.9 ± 1.1 ^c^	52.7 ± 2.0 ^b^
a*	3.5 ± 0.1 ^d^	3.3 ± 0.1 ^d^	1.8 ± 0.1 ^c^	-2.1 ± 0.1 ^a^	1.2 ± 0.2 ^b^
b*	15.3 ± 0.1 ^b^	13.6 ± 0.3 ^a^	16.4 ± 0.3 ^c^	20.6 ± 0.6 ^e^	18.3 ± 0.8 ^d^

EVP15-2(T), pasta from blend 85% Semolina 2 and 15% EVP extruded with Teflon-coated die; EVP15-2(B), pasta from blend 85% Semolina 2 and 15% EVP extruded with bronze die; EVF10, pasta from blend 90% Semolina 1 and 10% EVF extruded with Teflon-coated die; PS, pasta from Semolina 2 extruded with Teflon-coated die; PWS, pasta from Wholegrain semolina extruded with Teflon-coated die. ^1^ At least 20% of the energy value of the food is provided by protein. ^2^ At least 6 g of fibre per 100 g of product. ^3^ At least 12% of the energy value of the food is provided by protein. ^4^ At least 3 g of fibre per 100 g of product. * Calculated by difference.

**Table 6:** Amino acid (g/100 g protein), chemical score (CS) and limiting amino acid of BSG ingredients and pasta.

Essential Amino Acids	EVP	EVF	EVP15-2	EVF10	Semolina-2	Wholegrain Semolina	Amino Acid Scoring Pattern (mg/g) [45]
Histidine	3.2 ± 0.14	3.4 ± 0.11	3.3 ± 0.04	2.6 ± 0.15	2.5 ± 0.04	3.2 ± 0.28	16
Isoleucine	3.4 ± 0.02	4.1 ± 0.02	3.6 ± 0.12	3.6 ± 0.26	3.7 ± 0.35	4.2 ± 0.37	30
Leucine	8.9 ± 0.10	5.6 ± 0.08	7.2 ± 0.08	6.8 ± 0.32	7.7 ± 0.58	9.1 ± 0.74	61
Lysine	3.5 ± 0.14	3.4 ± 0.11	2.5 ± 0.05	2.3 ± 0.12	2.1 ± 0.01	2.4 ± 0.01	48
Methionine + Cysteine	3.4 ± 0.07	3.5 ± 0.22	3.6 ± 0.10	3.6 ± 0.21	3.5 ± 0.19	3.2 ± 0.20	23
Phenylalanine + Tyrosine	9.6 ± 0.22	12.0 ± 0.52	9.4 ± 0.20	8.6 ± 0.43	8.6 ± 0.61	9.8 ± 0.03	41
Threonine	3.7 ± 0.08	4.1 ± 0.01	3.1 ± 0.08	3.0 ± 0.12	2.9 ± 0.13	2.9 ± 0.14	25
Valine	5.3 ± 0.17	6.5 ± 0.10	5.2 ± 0.15	5.0 ± 0.23	5.2 ± 0.21	5.7± 0.12	40
Chemical Score (CS)	72	71	52	46	43	50	
Limiting Amino acid	Lysine	Lysine	Lysine	Lysine	Lysine	Lysine	

**Table 7:** Optimum cooking time, cooking quality, and texture analysis of different pastas. Different letters in rows indicate statistically significant differences (*p* < 0.05).

	EVP15-2(T)	EVP15-2(B)	EVF10	Semolina-2	Wholegrain Semolina
OCT (min)	12′50″	13′30″	11′00″	13′00″	10′00″
Cooking quality					
Firmness	86 ± 5 ^a^	94 ± 4 ^a^	87 ± 3 ^a^	88 ± 4 ^a^	89 ± 6 ^a^
Starch release	90 ± 3 ^b,c^	93 ± 4 ^c^	81 ± 2 ^a^	84 ± 3 ^a,b^	77 ± 4 ^a^
Liveliness	95 ± 2 ^c^	94 ± 2 ^c^	88 ± 3 ^b^	85 ± 2 ^b^	73 ± 2 ^a^
Total score	90 ± 4 ^a,b^	94 ± 6 ^b^	89 ± 4 ^a,b^	84 ± 5 ^a,b^	80 ± 2 ^a^
Final judgement	Excellent	Excellent	Excellent	Excellent	Good
Texture analysis					
Firmness (g)	560 ±15 ^a,b^	584 ±18 ^b^	529 ± 23 ^a^	581 ± 20 ^b^	515 ± 20 ^a^
Tensile strength	31 ± 6 ^a^	29 ± 2 ^a^	23 ± 6 ^a^	27 ± 6 ^a^	33 ± 7 ^a^
Flexure (g) (uncooked pasta)	47 ± 3 ^a^	62 ± 7 ^b^	42 ± 5 ^a^	52 ± 3 ^a,b^	48 ± 8 ^a^

EVP15-2(T), pasta from blend 85% Semolina 2 and 15% EVP extruded with Teflon-coated die; EVP15-2(B), pasta from blend 85% Semolina 2 and 15% EVP extruded with bronze die; EVF10, pasta from blend 90% Semolina 1 and 10% EVF extruded with Teflon-coated die; PS, pasta from Semolina 2 extruded with Teflon-coated die; PWS, pasta from Wholegrain semolina extruded with Teflon-coated die.

## Data Availability

The data presented in this study are available on request from the corresponding author.
